# Primitive sarcoma of the breast: new insight on the proper surgical management

**DOI:** 10.1186/s13046-015-0190-1

**Published:** 2015-07-30

**Authors:** Vittorio Pasta, Massimo Monti, Michela Cialini, Massimo Vergine, Paolo Urciuoli, Annunziata Iacovelli, Silvio Rea, Valerio D’Orazi

**Affiliations:** Department of Surgical Sciences, “Sapienza” University of Rome, Viale Regina Elena 324, 00161 Rome, Italy; Department of General Microsurgery and Hand Surgery, “Fabia Mater” Hospital, Via Olevano Romano 25, 00171 Rome, Italy; Department of Gynecology, “Fabia Mater” Hospital, Via Olevano Romano 25, 00171 Rome, Italy; Department of Surgical Oncology, University of L’Aquila, Via Vetoio località Coppito, 67100 L’Aquila, Italy

**Keywords:** Primitive breast sarcoma, Surgery, Mastectomy, Quadrantectomy, Skip metastases

## Abstract

**Background:**

Primitive sarcoma of the breast is a rare and challenging disease at high risk of recurrence and with poor prognosis. There are controversies in the diagnosis and management of such solid tumor due to its rarity and heterogeneity. This sarcoma is poorly responsive to both chemotherapy and radiotherapy, thus, surgery is the first and most important therapeutic approach. However, given the rarity of this type tumor it has not be possible to standardize unique guidelines for the proper surgical strategy to adopt. Here, we performed a retrospective study of new 10 cases of primitive sarcoma of the breast that underwent either mastectomy or a more conservative quadrantectomy, in the attempt to better standardize correct surgical indications.

**Methods:**

Ten new cases of primitive sarcoma of the breast were registered between 2002 and 2012 and constituted the study group. They underwent either mastectomy or quadrantectomy and the clinical, prognostic and survival characteristics after surgery were analysed.

**Results:**

Within the group of patients treated with mastectomy, two had metastasis and died because of that. Among the five patients treated with quadrantectomy four are alive and free of disease after 3 to 5 years follow-up, while the patient with sarcoma arising in pregnancy, although is still alive, developed lung metastases four years after surgery.

**Conclusions:**

The first and most important therapeutic approach to primary sarcomas of the breast is surgical which has the purpose to achieve radical tumor excision to prevent local recurrence and skip metastases. However, given the rarity of the condition and the consequent small number of cases in this, like in similar studies, it is not possible to draw any definitive conclusions and further studies with larger numbers are necessary. However it would appear that performing a larger procedure such as mastectomy rather than performing a more limited one such as a quadrantectomy, has no advantage in terms of overall prognosis.

## Introduction

Primary breast sarcoma (PBS) is a rare malignancy that originates from the mesenchymal tissue of the mammary gland. The variety of cells present in this tissue, such as fat cells, muscle cells, or endothelial cells, accounts for the heterogeneity of the histological types encountered: liposarcomas, angiosarcomas, and leiomyosarcomas. PBS represents <1 % of all primary breast malignancies and <5 % of all sarcomas [1–4]. PBS is a typically female disease that can rarely occur in male patients. Its natural history does not significantly differ from that of sarcomas of other anatomical sites, except for the angiosarcoma which has a more severe prognosis [5]. PBS can sometimes be diagnosed too late even in patients undergoing screening visits, due to the initial characteristics of this tumor that can mimic benign lesions, such as fibroadenoma or phyllodes tumor [6–8] and a correct diagnosis is important for planning efficacious surgery treatments. Five years overall survival (OS) does not exceed 55 % and it is influenced by several factors such as tumor localization and dimension, histological features and resection margin status [9–14].

Due to the rarity of the PBS, it appears difficult to develop univocal guidelines both for the surgical treatment and for any subsequent specific therapy for the breast [9]. Thus, most of the published studies are case reports or studies on small groups of patients. PBS, like all sarcomas, is poorly responsive both to chemotherapy and radiotherapy, although the role of adjuvant radiation or chemotherapy is still unclear in light of the limited size of study reports [7, 15, 16]. The first and most important treatment for PBS is surgery which has the purpose to obtain radical excision of the tumor, with negative margins (R0) and to avoid local tumor recurrence linked to the presence of possible skip metastases [17, 18]. A correct surgical approach is important in many tumors, as also shown by our studies [19]. In order to standardize the surgical approach for limb sarcomas, in 1983 Enneking proposed different types of surgery: 1) *intralesional* or contaminated surgery, when the tumor is sectioned or broken during the surgical excision; 2) *marginal* surgery when the tumor is completely removed following the margin of psudocapsule, although skip metastases can occur; 3) *wide* surgery, when the tumor is completely removed along with a clear layer of normal peritumoral tissue; and 4) *compartmental* surgery, when the anatomical compartment site of the tumor is completely removed [20, 21]. Compartmental treatment or wide excision are usually considered sufficiently curative in sarcoma management, with resection margins of at least 1–2 cm of normal peritumoral tissue to avoid skip metastasis [21–24].

In the attempt to better standardize correct surgical indications for PBS, we performed a retrospective review of 10 new cases of PBS registered from 2002 to 2012 that underwent either mastectomy or a more conservative quadrantectomy in our Surgical Departments, and analyzed their clinical, prognostic and survival characteristics after surgery.

## Materials and methods

### Study population

Patients with PBS were treated in the Department of Surgical Sciences, Sapienza University of Rome and in the Department of Gynecology, “Fabia Mater” Hospital in Rome, Italy, between 2002 and 2012. A total of 10 cases, 9 female and 1 male, were reported in the study. Clinical characteristics, including treatment strategies (i.e., type of surgery, radiotherapy and chemotherapy), histological type, and tumor size were retrospectively analyzed from the medical records. The follow-up information was acquired from the database, the medical records and contact with the patients or their families. Patients were classified into two groups (five patients/each) based on the type of the performed surgery: in the first group the patients (including the only male) were treated with mastectomy; in the second group the patients were treated with the more conservative quadrantectomy (Table 1).

**Table 1:** Patients, tumor type, surgical treatment and adjuvant therapies

No.	Sex	Age years	Tumor type	Surgical treatment	Adjuvant treatment
1	F	46	Stromal sarcoma	Mastectomy	chemo + radio
2	F	33	Angiosarcoma	Mastectomy	chemo + radio
3	F	41	Phylloides tumor	Mastectomy	chemo + radio
4	F	73	Pleomorphic sarcoma	Mastectomy	chemo + radio
5	M	69	Liposarcoma	Mastectomy	chemo + radio
6	F	45	Phylloides tumor borderline	Quadrantectomy	N/A
7	F	51	Phylloides tumor low grade	Quadrantectomy	N/A
8	F	63	Chondrosarcoma	Quadrantectomy	chemo + radio
9	F	64	Epithelial/mesenchymal tumor	Quadrantectomy	N/A
10	F	43	Sarcoma in phylloides tumor	Quadrantectomy	N/A

All participants provided written informed consent before participating in this study.

The study was carried out according to the Helsinki Declaration.

## Results

### Clinical and surgical data

Overall, 10 patients constituted the study group. They were 9 female and 1 male with median age at diagnosis of primary carcinoma of 52.8 years. One patients was <40 years, 5 patients were <60 years, and 4 patients were >60 years at diagnosis (Table 1). The histological analyses showed different istotypes within the two surgical treated groups (Table 1). Tumours were mainly localized in the right chest (7/10) and the size was described by the pathologist for all patients with a median of about 101.6mm (range 15 to 160 mm); a mitosis count was performed for all patients showing 50% up to ten mitoses per field; six out of 10 patients presented severe cellular atypia (Table 2).

**Table 2:** Tumors characteristics

Histological type	Diameter (mm)	Surgery	Mitotic index	Cellular atypia
Stromal sarcoma	60	simple mastectomy	2-5 %	yes
Angiosarcoma	60	total right mastectomy	55 %	yes
Phylloides tumor	150	total right mastectomy removal of right chest relapse	high	yes
Pleomorphic sarcoma	160	total left mastectomy	80 %	yes
Liposarcoma	150	total right mastectomy	--	no
Phyllodes tumor borderline	160	Quadrantectomy of the right upper outer quadrant	2 %	no
Phyllodes tumor low grade	100	Quadrantectomy of the right upper outer quadrant	<2 %	no
Chondrosarcoma	60	Quadrantectomy of the right upper outer quadrant	7 %	yes
Epithelial/mesenchymal tumor	150	Quadrantectomy of the right upper outer quadrant and axillary sentinel lymphonode removal	77 %	yes
Sarcoma in phylloides tumor	15	Quadrantectomy of the left upper outer quadrant	high	yes

In the first group, treated with mastectomy, the five patients were affected by, respectively, stromal sarcoma, angiosarcoma, phyllodes tumor with a high degree of malignancy, pleomorphic sarcoma, and liposarcoma (Table 1). The first three patients underwent total mastectomy after a previous not adequate lumpectomy in a different Hospital that left residual tumor on the margins of resection, as evaluated by histologic examination. The patient with phyllodes tumor with a high degree of malignancy underwent mastectomy because the size of tumor (about 150 mm of diameter) while the patient with liposarcoma (the only male) could not undergo conservative surgery due to the lack of available margins. The patients undergoing mastectomy, after a not adequate surgery, were subsequently treated with radiotherapy (50 Gy preoperative - 60 Gy postoperative in 30 fractions) and adjuvant chemotherapy (Epirubicin 60 mg/m2 day 1, 2; Ifosfamide 3g/m2 day 1, 2, 3; Mesna 3g/day 1, 2, 3, for 5 cycles).

**Fig. 1:**
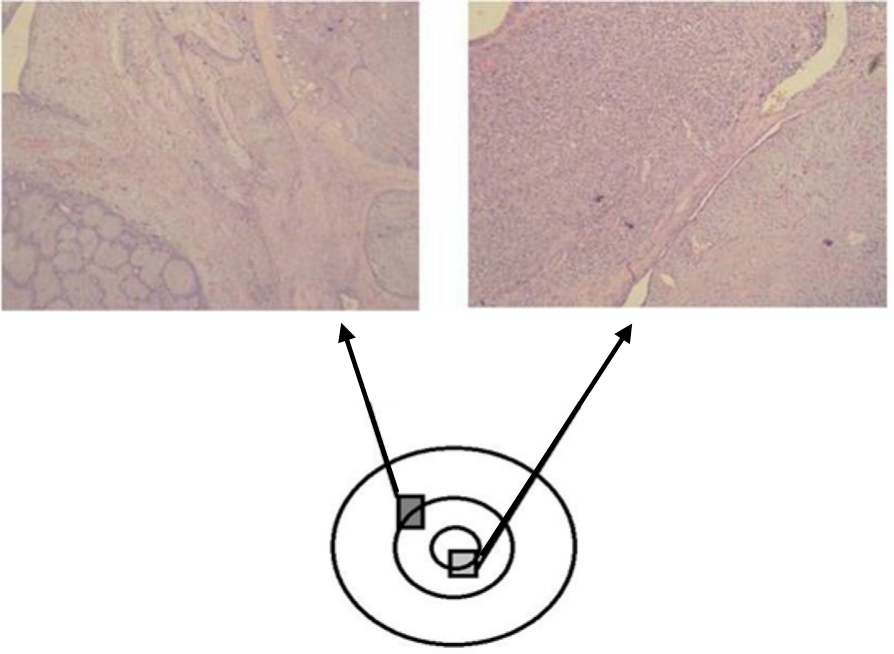
Photomicrograph showing breast sarcoma with unusual concentric lesion with sarcomatous tissue in the center, phyllodes tumor in the intermediate layer and tissue with histological features of fibroadenoma outside (magnification, x40)

In the second group, treated with more conservative quadrantectomy, the patients were affected by, respectively, borderline phyllodes tumor, chondrosarcoma, mixed epithelial/mesenchymal tumor, phyllodes tumor with low grade of malignancy, and sarcoma (Table 1). This last one was diagnosed in a pregnant patient and presented an unusual concentric lesion with sarcomatous tissue in the center, phyllodes tumor in the intermediate layer and tissue with histological features of fibroadenoma outside (Fig. 1). Among the patients undergoing quadrantectomy only the condrosarcoma patient was treated with radiotherapy (60 Gy in 30 fractions) and adjuvant chemotherapy (Epirubicin 60 mg/m2 day 1, 2; Ifosfamide 3g/m2 day 1, 2, 3; Mesna 3g/day 1, 2, 3, for 3–5 cycles).

### Survival data

To try to standardize general surgical indications for PBS we next analysed overall survival (OS) as the time from the surgery until death by any cause (Table 3). Among the five patients treated with mastectomy, the one with pleomorphic sarcoma died 1 year after surgery because the appearance of distant metastases while the patient with stromal sarcoma died 6 years after surgery, despite the more demolitive surgery performed. The other three patients treated with total mastectomy after lumpectomy underwent local recurrence of the disease and needed a new surgery, however, after 4 to 5 years follow-up they are free of disease.

**Table 3:** Patients, tumor type, and overall survival (OS)

No.	Tumor type	Surgical diagnosis	OS	Free of disease
1	Stromal sarcoma	Mastectomy	6 years	deceased
2	Angiosarcoma	Mastectomy	4 years	yes
3	Phylloides tumor	Mastectomy	5 years	yes
4	Pleomorphic sarcoma	Mastectomy	1 years	deceased
5	Liposarcoma	Mastectomy	4 years	yes
6	Phylloides tumor borderline	Quadrantectomy	5 years	yes
7	Phylloides tumor low grade	Quadrantectomy	5 years	yes
8	Chondrosarcoma	Quadrantectomy	5 years	yes
9	Epithelial/mesenchymal tumor	Quadrantectomy	3 years	yes
10	Sarcoma in phylloides tumor	Quadrantectomy	5 years	metastases

**Fig. 2:**
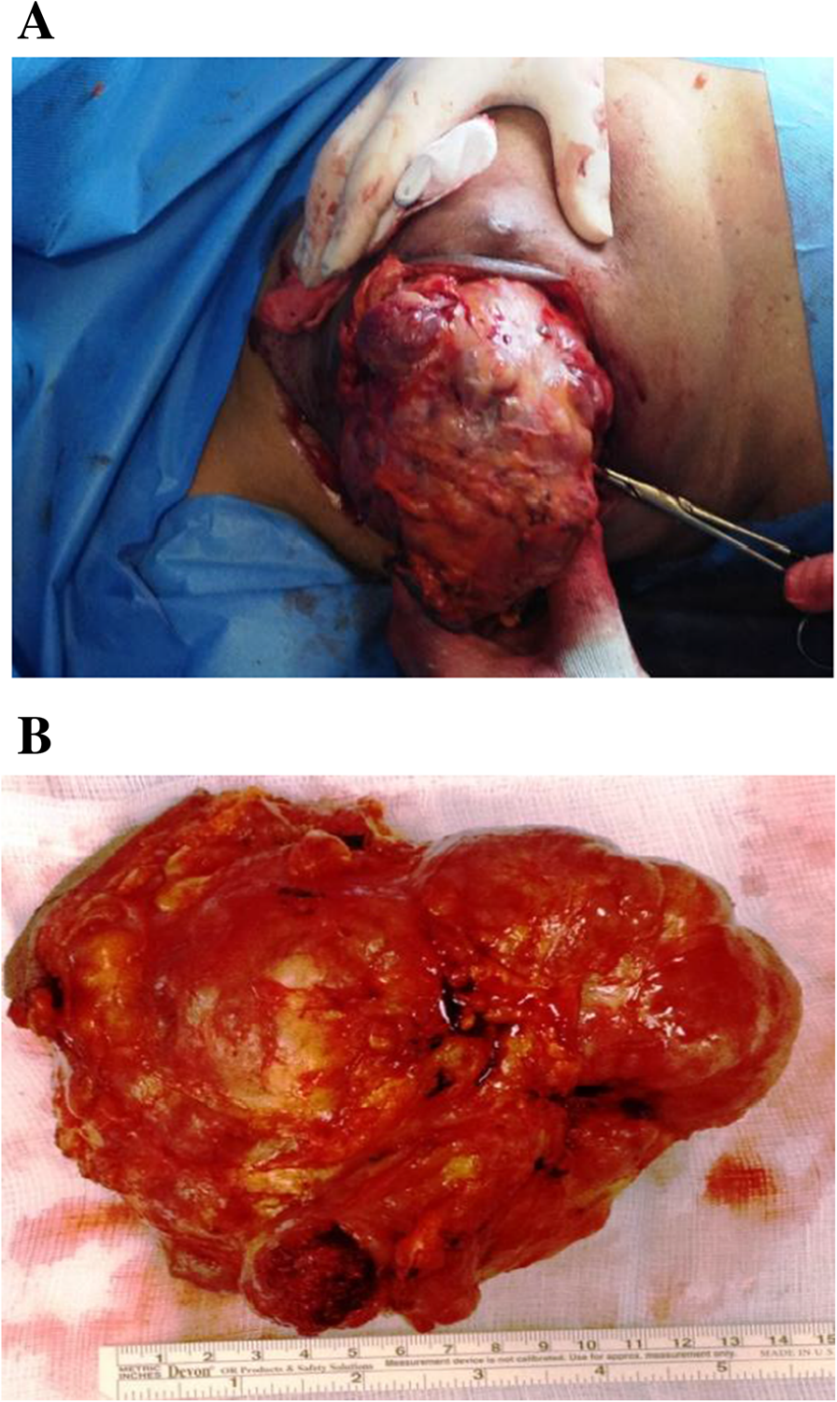
(**a**), (**b**) Macroscopic aspects of borderline phyllodes tumor

Among the five patients treated with the more conservative quadrantectomy four are alive and free of disease after 3 to 5 years follow-up, including the borderline phyllodes tumor patient, despite the large size of the tumor compared to the breast (Fig. 2), the chondrosarcoma patient, the mixed epithelial/mesenchymal metaplastic carcinoma patient and the phyllodes tumor with low grade malignancy patient. The mixed epithelial/mesenchymal metaplastic carcinoma patient, due to dual nature of the tumor, underwent outer quadrantectomy of the right breast with equatorial enlargement of the distal and proximal margins and removal of the sentinel lymph node with mixed reactive hyperplastic chronic lymphadenitis. Different was the course of the sarcoma arising in pregnancy, whereas excision of the lesion (with local anesthesia for gestational status of the patient) was followed by a subsequent further enlargement of resection margins, considering the final histological examination. Four years after surgery the patient developed lung metastases and is still alive underlying the importance of a correct surgical approach since the beginning (in this case, surgery was influenced by the pregnancy status of the patient).

## Discussion

Sarcoma of the breast is a very rare disease which represents <1% of breast cancers and <5% of all location sarcomas [15, 25]. For this reason, it is difficult to identify a specific treatment for these patients. There is widespread acceptance that surgical resection should be the first modality of treatment for breast sarcoma. However, it is still unclear which would be the surgical treatment with the best outcome for the patients [17, 18].

The purpose of surgery is excellent local control and for many years, mastectomy was regarded as the gold standard. The choice of a more or less demolitive surgical intervention (respectively, mastectomy and quadrantectomy) is based on the possibility to obtain adequate margins of healthy peritumoral tissue which would ensure both the complete removal of the tumor and the excision of possible skip metastases, that unlike what happens in other tumors, including melanoma, are not detectable by instrumental examinations [22–26]. To reach this goal, it is particularly important to evaluate the tumor/breast size ratio before surgery in order to properly perform a correct planning of the surgical strategy which could include even conservative surgery without compromising the prognosis [27]. Thus, we showed here that, despite the large size of the tumor compared to the breast (Fig. 2), the borderline phyllodes tumor patient was treated with the more conservative quadrantectomy that allowed to keep the patient still alive and free of disease after 5 years follow-up.

About the two patients undergoing mastectomy and that subsequently died of recurrent disease, it should be highlighted that the first case was a reoperation after inadequate excision previously performed elsewhere, while in the second case the diameter of the tumor (160 mm) and the tumor/breast size ratio did not allow another type of surgical approach. Among the patients treated with quadrantectomy, it was particularly interesting the case of the pregnant woman with “concentric” lesion (center sarcoma, intermediate layer phyllodes, outside fibroadenoma). This particular situation occurred in pregnancy did suggest the possible malignant evolution (from center to the periphery) of mesenchymal lesions originally benign, even if the observation of a single case allows us to only assume this type of evolution. Some studies show significantly increased OS after total mastectomy as compared with conservative surgery in PBS patients [8]. In contrast, other studies have demonstrated no significant advantage to mastectomy in comparison with wide local excision [1, 16, 28–30]. This is similar to our observation on the treatment of PBS patients.

Furthermore, local recurrence rate varies according to the location, the histological type and technical possibilities to perform a wide or compartmental excision [31]. Surgery is the first and most effective treatment for these tumors and indeed, with resection margins of less than 1 cm, the local recurrence rate is around 13%, while with resection margins larger than 1 cm local recurrence rate is around 0% [11, 32], since with wide resection margins the so-called skip metastasis are removed en bloc with the tumor. Therefore, surgeons must pay particular attention to the characteristics of the resection margins [7, 33, 34]. Importantly, only for angiosarcomas resection margins must be at least 3 cm of healthy peritumoral tissue, while for all other sarcomas of the breast a margin of 1 cm is considered sufficiently curative [15].

Mastectomy for mesenchymal neoplasms should not normally be performed with axillary lymphadenectomy, since these tumors mainly spread by blood vessels; the axillary lymphadenectomy may expose the patient to increased morbidity with no significant increase of disease-free survival and overall survival rate. Conversely for mixed tumors, lymphadenopathy or for the tumors along the axillary fold, sentinel lymph node biopsy can be considered [27].

Thanks to core-byopsy examination, we were actually able to identify both the degree and the histological type of the tumors, and we could better plan the preoperative surgical approach to sarcomas [31]. Furthermore, the new concepts about the embryonic development of the breast have redefined the idea of surgical anatomical compartment [35]. Indeed, thanks to recent studies on embryonic development of the mammary gland, a separate origin of the various glandular segments or lobules has been hypothesized and, therefore, the removal of an anatomical segment or quadrant can be considered as the resection of the corresponding anatomical compartment (compartmental surgery) [35].

After surgery, depending on the risk of relapse (related to the diameter, location and grade of the tumor) adjuvant radiation therapy may be indicated, possibly associated with chemotherapy. Radiotherapy (preoperative 50 Gy, postoperative 60 Gy) is really important in the treatment of sarcomas, since it allows, if used in combination with surgery and also for conservative surgery, to achieve results comparable to those obtained with radical, demolitive surgery. Chemotherapy (Epirubicin 60 mg/m^2^ day 1, 2; Ifosfamide 3 g/m^2^ day 1, 2, 3; Mesna 3 g/day 1, 2, 3, for 3–5 cycles) may represent advantage for patients with high-risk lesions [24].

In summary, in literature, we did not find that mastectomy has a better chance of long-term survival for sarcomas of the breast compared to wide excision or to quadrantectomy, while the simple lumpectomy has a more severe prognosis [27]. In our experience, the histological features and the correct first surgical approach influenced the prognosis more than the size of the tumor, and obviously, early diagnosis is fundamental, as indeed for all cancers.

## Conclusions

Sarcomas of the breast, like all sarcomas, are poorly responsive both to chemotherapy and radiotherapy and, therefore, surgery has the most important role. Concerning the prognosis, surgery has the purpose to obtain a radical excision (R0 margins), avoiding local recurrence linked to the presence of possible "skip metastases". As highlighted in some of our cases, surgery is not effective in preventing distant metastases if they were already present at the time of the operation, even if not still diagnosable. Wide demolitive surgery (based on old concept of anatomical compartment) not always positively influences the prognosis, and even our study shows that the prognosis is mainly influenced by aggressiveness of the tumor, evaluated with a preoperative biopsy, correct first surgical approach and treatment, and early diagnosis. Furthermore, we do not have to underestimate functional, aesthetic and psychological aspects that may influence the quality of patients life after a more demolitive surgery, sometimes without a real improvement of disease-free period and overall survival.

Recent embryological studies confirm segmental/lobular origin of breast cancer. Thanks to these new informations we have now a different concept of anatomical compartment for sarcomas of the breast and, when possible, we can choose to perform less demolitive operations (quadrantectomy) with the same results of a more aggressive but not more useful surgery. The adjuvant radiotherapy following conservative surgery is surely useful in monitoring local recurrences, especially for larger and high grade tumors. The role of adjuvant chemotherapy is still controversial, and it may be indicated for more severe prognosis cancers.

## Abbreviations

PBS: primary breast sarcoma
Gy: Gray
OS: overall survival
g: grams
